# Identification of Isoliensinine as a Novel CCR5 Inhibitor for the Prevention of Skeletal Muscle Atrophy Through Virtual Screening and Experimental Validation

**DOI:** 10.3390/molecules31162837

**Published:** 2026-08-14

**Authors:** Taiqi Qu, Yujuan Chen, Yijia Zhang, Yuan Wang, Yixuan Li, Yanan Sun

**Affiliations:** 1 Key Laboratory of Functional Dairy, College of Food Science and Nutritional Engineering, China Agricultural University, Beijing 100083, China; sy20253314226@cau.edu.cn (T.Q.); b20253060775@cau.edu.cn (Y.Z.); 2 Department of Nutrition and Health, China Agricultural University, Beijing 100091, China; chenyj@cau.edu.cn (Y.C.); wy.foodsci@cau.edu.cn (Y.W.)

**Keywords:** skeletal muscle atrophy, C-C chemokine receptor type 5 (CCR5), Isoliensinine (ISO), pharmacophore-based virtual screening, molecular docking, molecular dynamics simulations

## Abstract

Age-related skeletal muscle atrophy (sarcopenia) poses a major public health challenge, emphasizing the need for safe and effective interventions. Our previous studies demonstrated that C-C chemokine receptor type 5 (CCR5) is a key therapeutic target for skeletal muscle atrophy, as its activation by C-C motif chemokine ligand 11 (CCL11) promotes the dissociation and degradation of the structural protein α-actin, ultimately contributing to muscle loss. To identify potential CCR5 inhibitors, a database of 7860 natural alkaloids was constructed for pharmacophore-based virtual screening using the CCR5–Maraviroc crystal structure. Screening yielded 789 candidates, and subsequent batch molecular docking analysis identified Isoliensinine (ISO), a lotus seed alkaloid, as a potential CCR5 inhibitor with low binding energy (−10 kcal/mol) and stable hydrogen bonding interactions with Glu283 and Tyr251. Molecular dynamics simulations further confirmed the structural stability of the ISO–CCR5 complex. Molecular dynamics simulations further confirmed the structural stability of the ISO-CCR5 complex. In vitro, ISO dose-dependently inhibited CCL11-induced CCR5 activity (IC_50_ = 1.314 μM) with low cytotoxicity in C2C12 myotubes, and markedly alleviated CCL11-induced myotube atrophy by suppressing CCR5 activation and the upregulation of the muscle atrophy–related markers MAFbx and MuRF1. These findings provide preliminary evidence for ISO as a potential CCR5-targeting candidate for further investigation in sarcopenia.

## 1. Introduction

Age-related loss of skeletal muscle mass and function, known as sarcopenia, is a progressive musculoskeletal disorder that contributes substantially to disability, functional decline, and reduced quality of life in older adults [1,2]. The proportion of lean body mass typically decreases from approximately 50% of total body weight in young adults to nearly 25% in individuals over 80 years of age [1,3]. Consequently, up to 42% of individuals over 60 years of age experience difficulties in performing activities of daily living, such as rising from a chair, while many also exhibit reduced muscle strength and varying degrees of physical disability [4]. These impairments are associated with increased risks of falls, institutionalization, comorbidities, and premature mortality [5]. Therefore, the development of effective interventions for sarcopenia remains a major public health priority in aging societies.

Aberrant activation of the senescence-associated secretory phenotype (SASP) is recognized as a key molecular feature of skeletal muscle aging and sarcopenia [6,7]. SASP comprises a diverse array of secreted bioactive factors, including chemokines, pro-inflammatory cytokines, and growth factors [6,8]. Through receptor-mediated signaling, these factors promote chronic inflammation and structural protein turnover in aging skeletal muscle, contributing to protein depolymerization, degradation, and ultimately the disruption of muscle homeostasis [9]. C-C chemokine receptor type 5 (CCR5), a member of the G protein-coupled receptor (GPCR) superfamily, mediates intracellular signaling through heterotrimeric G proteins [10,11]. Emerging evidence indicates that CCR5 participates in the transmission of SASP-associated chemokine signals, including CCL3, CCL4, and CCL5, within aged muscle stem cells (MuSCs) and their surrounding cellular microenvironment, thereby facilitating inflammatory responses, cellular senescence, and muscle degeneration [12]. Furthermore, pharmacological or genetic inhibition of CCR5 has been shown to alleviate skeletal muscle atrophy by suppressing oxidative stress and restoring protein homeostasis [13]. Our unpublished observations suggest that C-C motif chemokine ligand 11 (CCL11) may promote α-actin depolymerization and degradation in aged skeletal muscle through a CCR5-dependent mechanism. Collectively, these findings identify CCR5 as a promising therapeutic target for the prevention and treatment of muscle wasting. However, clinically approved small-molecule inhibitors targeting CCR5 for sarcopenia intervention remain unavailable.

Structurally, CCR5 exhibits a canonical seven-transmembrane (7TM) helical architecture composed of seven transmembrane α-helices (TM1–TM7), extracellular and intracellular loops, and an extracellular N-terminal region involved in ligand recognition and receptor activation [14]. Recent structural studies have revealed that ligand recognition is mediated by coordinated interactions between the extracellular region and the transmembrane helical bundle, which together regulate CCR5 conformational states and signaling activity [14]. The crystal structure of CCR5 in complex with the antagonist maraviroc (MVC) provides an important structural framework for inhibitor development. Unlike endogenous chemokines that primarily engage extracellular recognition regions, MVC binds within a deep hydrophobic cavity formed by the transmembrane helices of CCR5 and stabilizes the receptor in an inactive conformation through an allosteric mechanism [15]. This binding pocket is mainly composed of residues from TM1, TM2, TM3, TM5, TM6, and TM7, including Tyr37, Trp86, Tyr108, Phe109, Tyr251, and Glu283, which contribute to ligand stabilization through hydrogen bonding, hydrophobic interactions, and electrostatic contacts [15]. Therefore, the structural features of the MVC-binding pocket provide a rational basis for pharmacophore modeling and structure-based identification of novel CCR5 ligands.

Natural products and food-derived bioactive compounds have attracted increasing attention as potential strategies for the prevention and management of sarcopenia. Among these compounds, alkaloids have been reported to exert protective effects through multiple mechanisms, including suppression of protein degradation, stimulation of protein synthesis, regulation of inflammatory responses, improvement of mitochondrial function, and maintenance of autophagic homeostasis [16,17,18]. For example, capsaicin, a naturally occurring alkaloid in chili peppers, has been shown to alleviate muscle atrophy by restoring the balance between protein synthesis and degradation through activation of the Akt/mTOR signaling pathway and by preserving autophagy–lysosome homeostasis [18]. Several CCR5 antagonists have been developed, among which maraviroc (MVC) is the only approved small-molecule CCR5 inhibitor. However, despite extensive efforts, many CCR5 antagonists have failed to achieve clinical translation due to limitations including insufficient efficacy or safety concerns, highlighting the continued need for novel CCR5-targeting compounds [19]. Previous studies have demonstrated the potential of computational approaches for CCR5 ligand discovery. For example, pharmacophore-based screening combined with molecular docking and molecular dynamics simulations has been applied to identify potential CCR5 inhibitors; however, comprehensive experimental validation, including inhibitory activity, cytotoxicity, and functional assays, remains limited [19]. In addition, structure-based virtual screening using CCR5 homology models has successfully identified nonpeptide CCR5 ligands, although the accuracy of ligand–receptor interaction prediction was constrained by the lack of experimentally determined receptor structures [20]. With the availability of the high-resolution CCR5–MVC crystal structure, structure-guided strategies incorporating experimentally defined ligand–receptor interactions provide new opportunities for more reliable CCR5 inhibitor discovery. Therefore, in this study, we developed a CCR5–MVC structure-based pharmacophore model and performed batch molecular docking to identify potential CCR5-binding natural alkaloids.

## 2. Results

### 2.1. Pharmacophore-Based Virtual Screening of CCR5 Inhibitors

**Figure 1:**
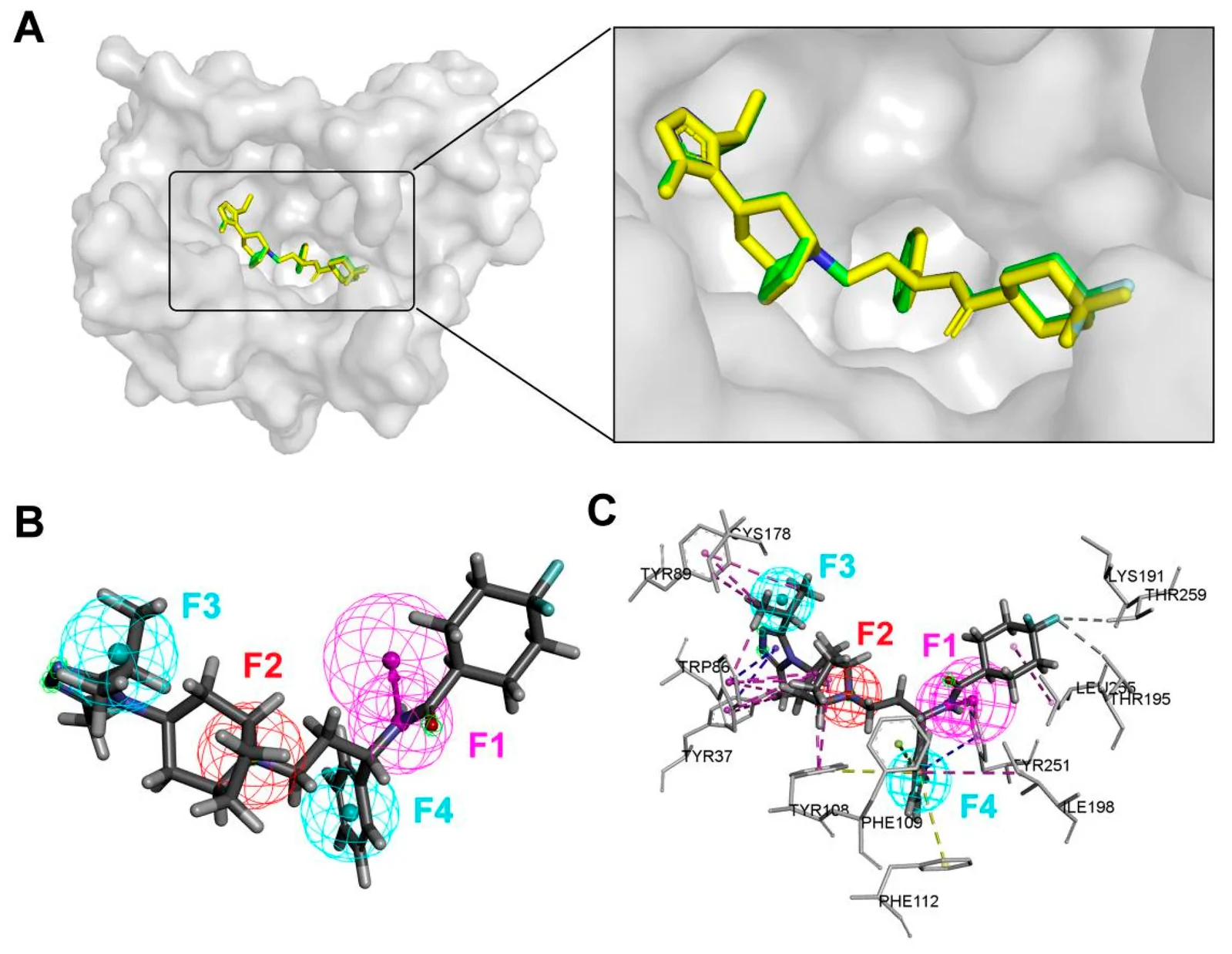
Pharmacophore features and molecular interactions of the CCR5–MVC complex. (**A**) Validation of docking protocol by comparison of the LibDock-predicted MVC pose with the crystallographic MVC conformation in the CCR5 binding pocket. The crystallographic MVC conformation (yellow) and LibDock-generated docking pose (green) showed excellent structural overlap. (**B**) Pharmacophore model based on CCR5-MVC co-crystal structure (F1 is a hydrogen-bond donor feature, F2 is an ionizable feature, and F3 and F4 are hydrophobic features). Pharmacophore features are represented by spheres. (**C**) Overlap of pharmacophore features with CCR5–MVC docking interactions. π–alkyl interactions are represented by the purple dotted line, π–π stacked interactions are represented by the blue dotted line, π–π T-shaped interactions are represented by the yellow dotted line, π–sigma interactions are represented by the green dotted line, and fluorine-mediated interactions are represented by the grey dotted line.

Maraviroc (MVC), a well-established CCR5 antagonist, was selected as the reference ligand for pharmacophore model construction [15]. The CCR5–MVC co-crystal structure (PDB ID: 4MBS) provides an experimentally validated binding mode. To verify the reliability of the docking protocol, the docking-derived MVC conformation was compared with the crystallographic ligand conformation. As shown in Figure 1A, the docked and experimental MVC conformations exhibited excellent agreement, with a heavy-atom RMSD of 0.196 Å, indicating that the docking procedure accurately reproduced the native CCR5–MVC binding mode. Therefore, the docking-derived MVC conformation was considered a reliable representation of the experimentally validated binding pattern and was used for subsequent pharmacophore model generation. As shown in Figure 1B, four typical pharmacophore features (called F1–F4) were generated, including one hydrogen bond donor feature (F1), one Ionizable feature (F2), and two hydrophobic features (F3 and F4). Molecular interaction analysis demonstrated that MVC formed multiple non-covalent interactions within the CCR5 binding pocket, including π–alkyl, π–sigma, π–π stacked, and π–π T-shaped interactions, which collectively contributed to stabilization of the receptor–ligand complex (Figure 1C). The Ionizable feature F2 corresponded to π–alkyl interactions formed between MVC and residues Tyr108 and Trp86. In addition, the hydrophobic features F3 and F4 were primarily associated with π–alkyl interactions involving Tyr89, Cys178, and Ile198, π–π stacked interactions with Tyr251, π–π T-shaped interactions with Phe112, and π–sigma interactions with Phe109. Based on the identified pharmacophore features (F1–F4), a CCR5 pharmacophore model was subsequently established and applied for docking-based virtual screening.

The small molecule database of 7860 alkaloids was established for virtual screening. A total of 1524 ‘hit’ compounds matching the pharmacophore features were obtained, as shown in Supplementary Table S2. 789 ‘hit’ compounds with fit values > 1 and RMSD < 2 were selected for further molecular docking.

### 2.2. Molecular Docking and Interaction Analysis

**Figure 2:**
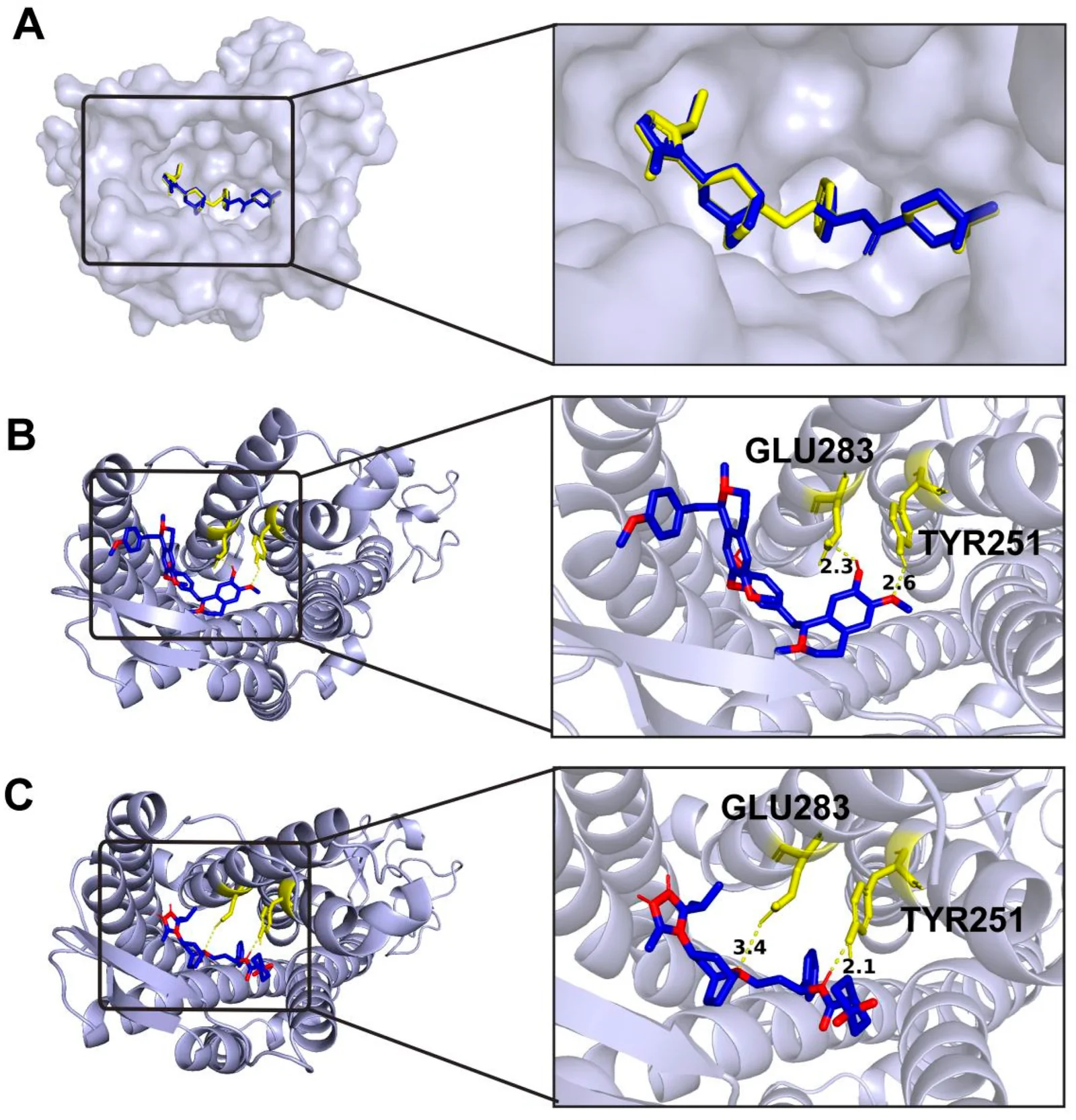
The X-ray crystal structure and the predicted docking poses of ISO and MVC at the CCR5 active site. (**A**) Validation of the molecular docking protocol by MVC redocking. The crystallographic conformation of MVC (yellow) was highly superimposable with the AutoDock Vina-redocked MVC pose (blue); (**B**) ISO docking pose; (**C**) MVC docking pose. The ligands are shown as sticks colored by chain (blue and red), and CCR5 is shown in light blue. Hydrogen bonds are represented by yellow dashed lines, while interacting residues are highlighted in yellow.

To validate the reliability of the molecular docking protocol, redocking analysis was initially performed using the co-crystallized ligand MVC. After removing MVC from the CCR5–MVC complex structure, MVC was redocked into the predefined binding pocket using identical docking parameters. As shown in Figure 2A, the redocked MVC pose was highly superimposable with the crystallographic MVC conformation, with an RMSD value of 0.209 Å. This excellent agreement between the predicted and experimental structures demonstrated the reliability of the established docking protocol for reproducing the native binding mode. Subsequently, molecular docking was performed to evaluate the binding affinity of 789 alkaloids toward the CCR5 binding pocket. All compounds were docked under identical conditions, and their docking scores are summarized in Supplementary Table S3. As shown in Table 1, the top 15 candidates with docking scores comparable to the reference CCR5 antagonist maraviroc (MVC, −10.8 kcal/mol) were further evaluated based on their binding modes, interactions with key CCR5 residues, and availability for experimental validation. These candidates exhibited comparable predicted binding energies ranging from −11.4 to −10.0 kcal/mol; however, only a subset displayed MVC-like interactions involving critical residues such as Glu283 and Tyr251. Among them, Hodgkinsine showed the lowest predicted binding energy (−11.4 kcal/mol) and shared key interactions with MVC, but its limited availability hindered further experimental evaluation. In contrast, isoliensinine (ISO) was selected for subsequent investigation due to its favorable accessibility, natural abundance, and ease of extraction from lotus seeds. ISO exhibited a comparable docking score (−10.0 kcal/mol), formed stable hydrogen bonds with key CCR5 residues including Glu283 and Tyr251, and exhibited a binding mode highly similar to that of the classical CCR5 antagonist MVC (Figure 2B,C). Overall, ISO was identified as a promising CCR5-binding candidate with favorable binding affinity and interaction characteristics.

### 2.3. Molecular Dynamics Simulations of ISO

**Figure 3:**
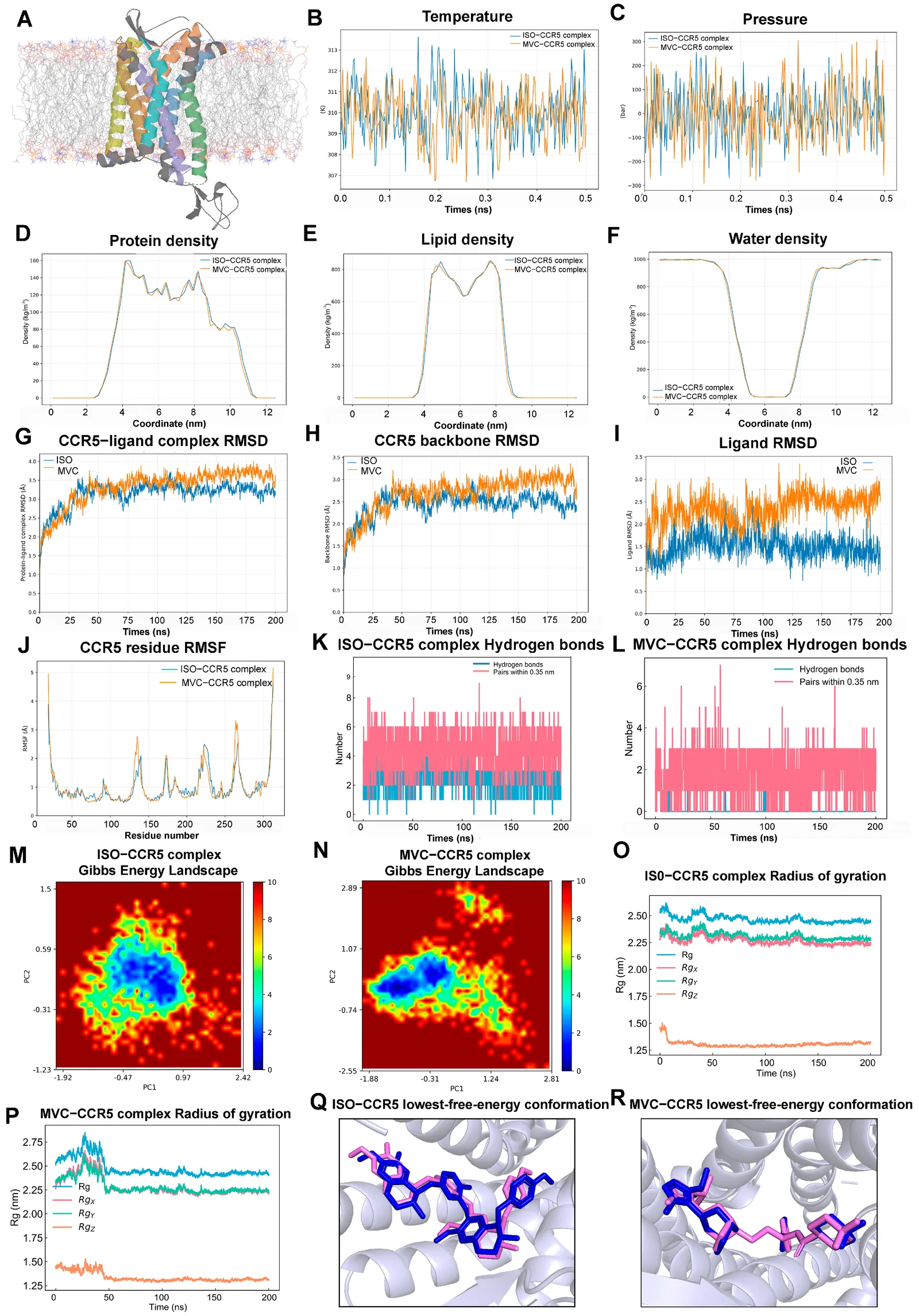
Molecular dynamics simulation of the ISO–CCR5 and MVC–CCR5 complexes. (**A**) Schematic representation of the constructed molecular dynamics system, showing CCR5 embedded in a POPC lipid bilayer and surrounded by the aqueous solvent environment. (**B**,**C**) Temperature and pressure profiles of the ISO–CCR5 and MVC–CCR5 systems during the sixth and final equilibration stage. (**D**–**F**) Protein, lipid, and water density distributions along the membrane normal. (**G**–**I**) Root-mean-square deviation profiles of the CCR5–ligand complexes, CCR5 backbones, and ligands relative to the receptor backbone during the 200 ns production simulations. (**J**) Residue-level root-mean-square fluctuation profiles of CCR5 in the two systems. (**K**,**L**) Numbers of hydrogen bonds for the ISO–CCR5 and MVC–CCR5 complexes. (**M**,**N**) Gibbs free-energy landscapes projected onto the first two principal components for the ISO–CCR5 and MVC–CCR5 systems, respectively; lower-energy regions are shown in blue and higher-energy regions in red. (**O**,**P**) Overall and directional radii of gyration of the ISO–CCR5 and MVC–CCR5 complexes, respectively. (**Q**,**R**) Superposition of the initial docking poses and lowest-free-energy conformations of ISO and MVC within the CCR5 binding pocket, respectively. The initial docking poses and lowest-free-energy conformations are shown in blue and magenta, respectively.

CCR5 was embedded in a POPC lipid bilayer and solvated to construct the membrane protein simulation system (Figure 3A). The ISO–CCR5 and MVC–CCR5 systems were then subjected to six sequential equilibration stages, followed by 200 ns production simulations. Temperature and pressure profiles from the sixth and final equilibration stage showed that both systems remained close to the target temperature of 310 K, with no systematic drift (Figure 3B,C). Although instantaneous pressure fluctuated substantially, it remained centered around the reference pressure of 1 bar without a sustained trend. In addition, the protein, lipid, and water density profiles along the membrane normal were highly similar between the two systems, indicating comparable receptor positioning, bilayer organization, and solvent distribution (Figure 3D–F). These results confirmed that both systems reached comparable thermodynamic and structural states before production simulation.

During the production simulations, both complexes underwent an initial structural relaxation and subsequently reached relatively stable conformational states. After the early adjustment phase, the CCR5 backbone RMSD in the ISO-bound system remained mainly within 2.3–2.7 Å, whereas the MVC-bound system showed higher values and gradually increased to approximately 3.0 Å at later stages (Figure 3H). The protein–ligand complex RMSD showed a similar pattern, with consistently lower deviations in the ISO–CCR5 system (Figure 3G). Likewise, ligand RMSD relative to the CCR5 backbone was lower for ISO, generally ranging from 1.2 to 1.7 Å, compared with approximately 2.0–2.8 Å for MVC (Figure 3I). These findings indicate that ISO maintained a more stable binding pose and induced smaller overall conformational deviations in CCR5.

The residue-level RMSF profiles were broadly similar between the two systems, indicating preservation of the overall dynamic pattern of CCR5 (Figure 3J). Most residues exhibited fluctuations below 1.0 Å, whereas greater mobility was observed at the N- and C-termini and in several loop regions. MVC induced higher fluctuations around residues 130–140 and 260–270, whereas ISO produced moderately greater mobility around residues 220–230, suggesting ligand-specific modulation of local CCR5 flexibility.

Hydrogen-bond analysis further distinguished the two complexes. ISO maintained approximately one to three hydrogen bonds with CCR5 throughout most of the simulation, occasionally increasing to four, whereas MVC formed fewer persistent hydrogen bonds (Figure 3K,L). ISO also exhibited more donor–acceptor pairs within 0.35 nm, indicating a more stable and persistent hydrogen-bonding network.

The Gibbs free-energy landscapes revealed distinct conformational distributions. The ISO–CCR5 system displayed a compact and continuous low-energy basin, consistent with a more restricted conformational ensemble (Figure 3M). In contrast, the MVC–CCR5 system exhibited a broader and more heterogeneous landscape with multiple separated low-energy regions, indicating greater conformational flexibility and transitions among metastable states (Figure 3N).

Both complexes maintained stable overall compactness. The ISO–CCR5 complex stabilized at an overall radius of gyration of approximately 2.44–2.50 nm after a modest initial adjustment (Figure 3O). The MVC–CCR5 complex showed larger fluctuations during the first 40–50 ns, including a transient increase to approximately 2.8 nm, before stabilizing at approximately 2.40–2.45 nm (Figure 3P). The directional components of the radius of gyration also reached stable plateaus in both systems.

Superposition of the initial docking poses with the lowest-free-energy conformations yielded ligand RMSD values of 1.024 Å for ISO and 0.604 Å for MVC (Figure 3Q,R). These low values indicate that both ligands largely retained their original binding orientations within the CCR5 pocket, although ISO underwent slightly greater local conformational adjustment. Overall, these results indicate that both complexes remained stable during the simulations, while ISO formed a more persistent and conformationally defined interaction with CCR5 than MVC.

### 2.4. Inhibitory Effects of ISO on CCR5 Expression and Cell Viability

**Figure 4:**
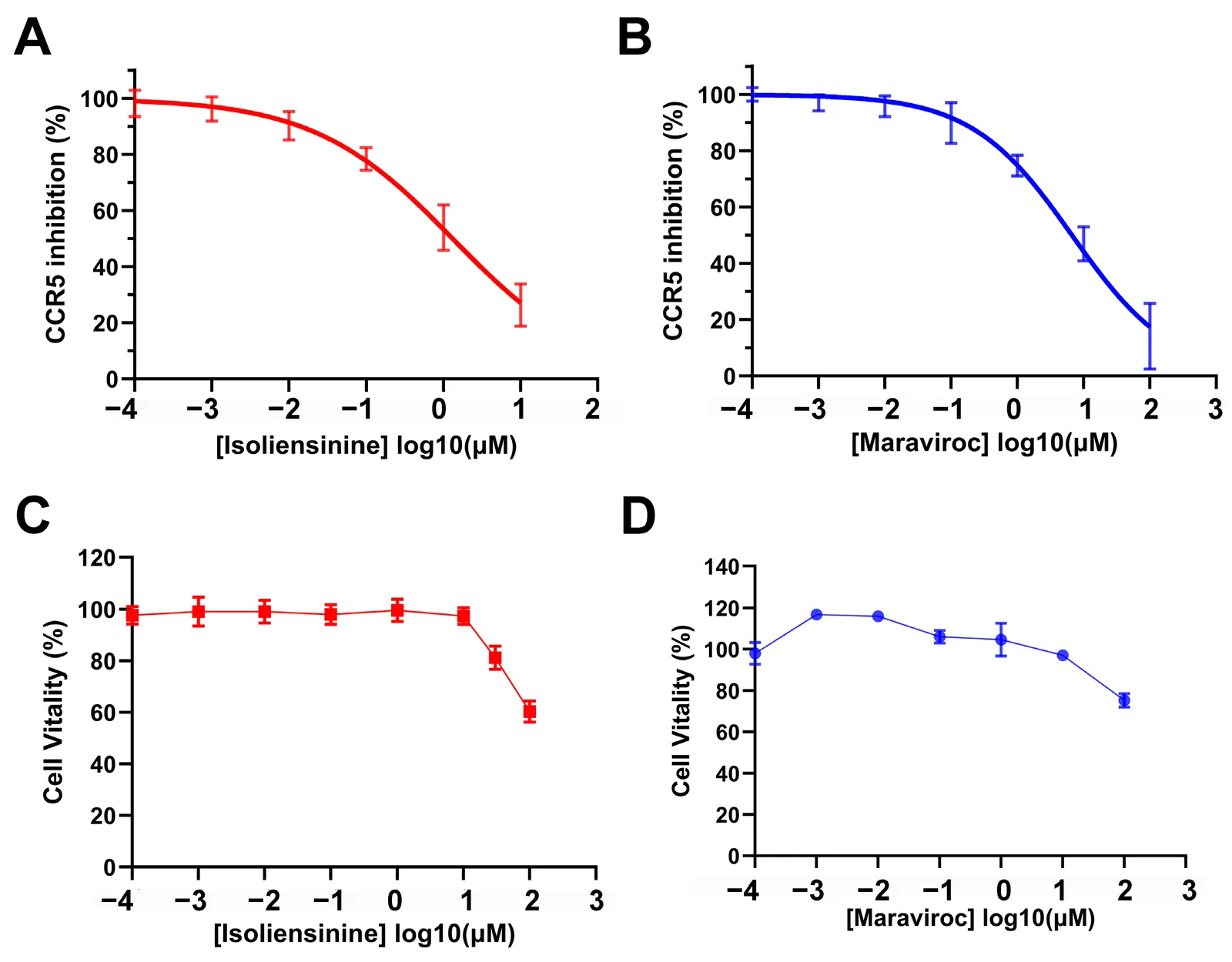
Concentration-dependent inhibition of CCR5 signaling by ISO and MVC and effects on C2C12 cell viability. (**A**,**B**) Concentration–response curves for inhibition of CCL11 (1 μM) signaling via receptor CCR5 in C2C12 cells by ISO and MVC. Receptor activation was measured using enzyme-linked immunoassay to detect production of cAMP. The cAMP concentration induced by treatment with 30 μM Forskolin (FSK) for 15 min was taken as the maximum cAMP concentration. (**C**,**D**) Dose-dependent effects of ISO and MVC on C2C12 cell viability measured by CCK-8 assay.

It is known that CCR5, as a G protein-coupled receptor, can be activated by CCL11, with this activation leading to a decrease in the downstream second messenger cAMP [21,22,23]. To validate the inhibitory effect of ISO on CCR5, we measured cAMP expression using a competitive enzyme-linked immunosorbent assay. As shown in Figure 4A, ISO inhibited CCR5 activity in a concentration-dependent manner. Increasing concentrations of ISO progressively attenuated the inhibitory effect of CCR5 on forskolin-stimulated cAMP production. The half maximal inhibitory concentration (IC_50_) of ISO on CCR5 protein expression induced by CCL11 was 1.314 [0.9266–1.890] μM, which is almost 1/5 of the IC_50_ (6.670 [4.728–9.411] μM) of MVC on CCR5 (Figure 4A,B; Table 2). This result indicates that ISO exhibits stronger inhibitory activity against CCR5 than MVC. To assess the cytotoxic effects of ISO, we performed a CCK8 assay using C2C12 cells. As shown in Figure 4C and Table 2, the half maximal cytotoxic concentration (CC_50_) of ISO in C2C12 cells was greater than 30 μM, significantly higher than its IC_50_. In contrast, MVC exhibited negligible cytotoxicity toward C2C12 cells (Figure 4D; Table 2). Overall, ISO demonstrates potent inhibitory activity against CCR5 with a favorable safety profile in C2C12 cells.

### 2.5. Ameliorative Effect of ISO on C2C12 Myotube Atrophy

**Figure 5:**
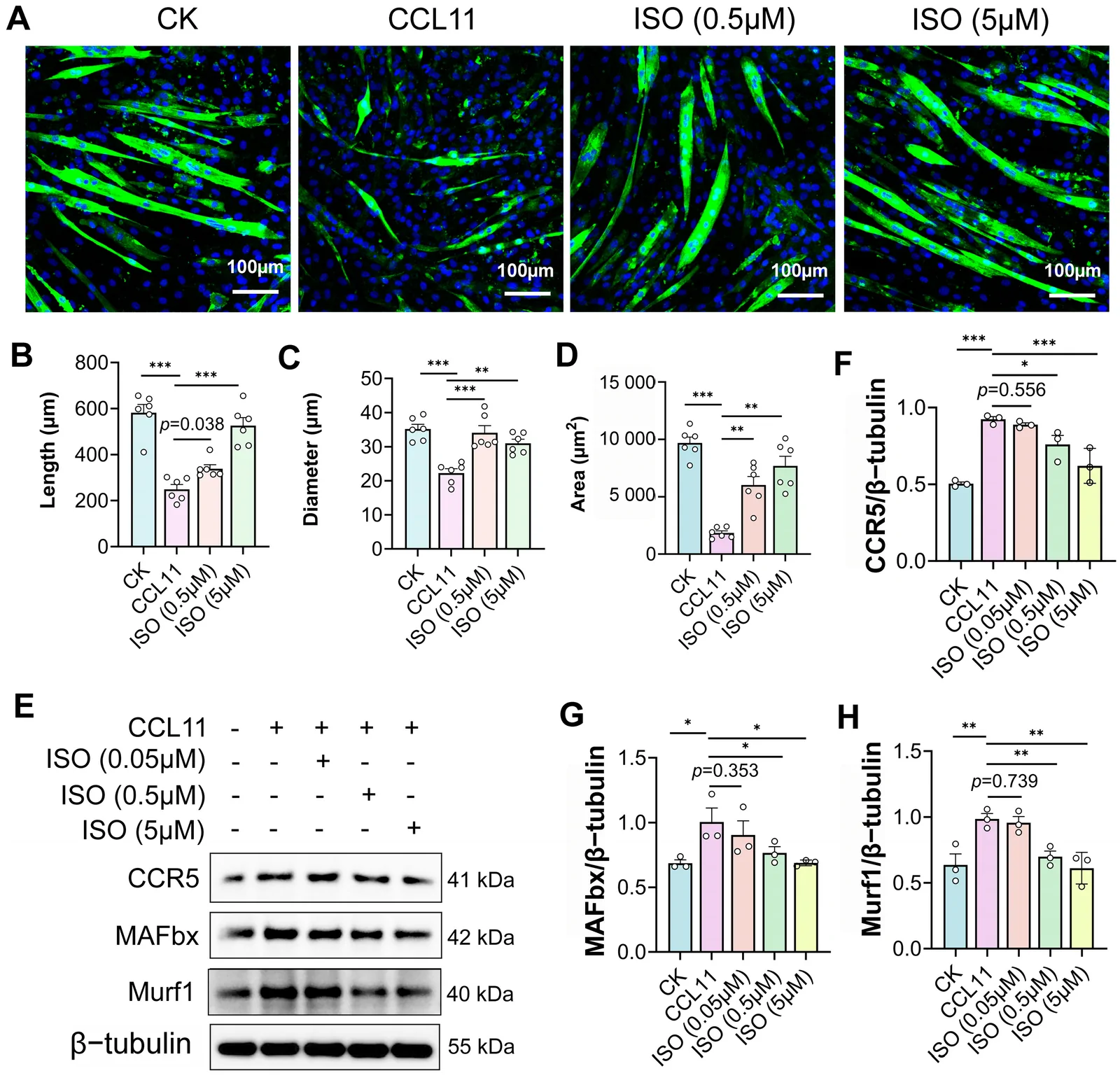
ISO protects C2C12 myotubes against CCL11-induced atrophy through activating CCR5. (**A**–**D**) MyHC immunostaining (magnification 10×; scale bar  =  100 µm) and quantification of myotubes. CK  =  control group, CCL11  =  CCL11 (1 μM) treatment group, ISO (0.05 μM)  =  ISO (0.05 μM) combined with CCL11 (1 μM) treatment group, ISO (0.5 μM)  =  ISO (0.5 μM) combined with CCL11 (1 μM) treatment group, ISO (5 μM) = ISO (5 μM) combined with CCL11 (1 μM) treatment group. (**E**–**H**) Western blot and quantification of CCR5, MAFbx and MuRF1. *, *p* < 0.05; **, *p* < 0.01; ***, *p* < 0.001.

C2C12 myotubes were treated with 1 μM CCL11 for 72 h to establish an in vitro model of muscle atrophy. Immunostaining of MyHC revealed that CCL11 treatment markedly reduced myotube length, diameter, and area to 42.84%, 63.32%, and 19.07% of the control levels, respectively (Figure 5A–D). Treatment with 0.5 μM ISO significantly attenuated the CCL11-induced reductions in myotube diameter and area, restoring them to 96.91% and 62.19% of the control levels, respectively (Figure 5A–D). In addition, 0.5 μM ISO partially restored myotube length to 58.24% of the control level. The protective effects of ISO were more pronounced at 5 μM, with myotube length, diameter, and area recovering to 90.40%, 88.08%, and 79.44% of the control levels, respectively (Figure 5A–D).

Western blot analysis further demonstrated that CCL11 treatment activated CCR5 signaling and significantly upregulated the expression of muscle wasting biomarkers MAFbx and MuRF1 (Figure 5E–H). Both 0.5 μM and 5 μM ISO markedly suppressed CCL11-induced CCR5 activation and the subsequent upregulation of MAFbx and MuRF1, whereas 0.05 μM ISO showed no significant protective effect (Figure 5E–H). Collectively, these findings demonstrate that ISO alleviates CCL11-induced atrophy in differentiated C2C12 myotubes through the inhibition of CCR5.

### 2.6. Predicted ADME Properties, Toxicity Profile, and Target Landscape of ISO

To further evaluate the pharmacological properties of ISO, its physicochemical characteristics, ADME properties, and drug-likeness were predicted using SwissADME. ISO exhibited a molecular weight of 610.74 g/mol, a topological polar surface area (TPSA) of 83.86 Å^2^, and two hydrogen-bond donors with eight hydrogen-bond acceptors (Supplementary Table S4). The predicted consensus LogP value was 5.20, indicating relatively high lipophilicity. ISO showed high gastrointestinal absorption but was predicted not to penetrate the blood–brain barrier. Drug-likeness analysis revealed only one Lipinski violation, attributable to the relatively high molecular weight of ISO, while no PAINS or Brenk structural alerts were identified, suggesting that ISO possesses acceptable medicinal chemistry characteristics with limited structural liabilities.

To compare the potential target profiles of ISO and the reference CCR5 antagonist MVC, SwissTargetPrediction analysis was performed. MVC showed a highly CCR5-focused predicted target profile, with CCR5 ranked as the top predicted target (probability = 1.00), consistent with its established pharmacological mechanism. In contrast, ISO exhibited a broader predicted target spectrum, with butyrylcholinesterase (BCHE) and several GPCR family members, such as dopamine receptors (DRD2 and DRD3), β2-adrenergic receptor (ADRB2), and 5-HT1A receptor (HTR1A), among the top-ranked predicted targets (Supplementary Table S5). These findings suggest that ISO may possess a distinct pharmacological profile compared with MVC. Although CCR5 was not ranked among the top predicted targets of ISO, this ligand-based prediction does not exclude ISO–CCR5 interaction, which was supported by molecular docking and functional validation.

To further assess the preliminary safety profile of ISO, in silico toxicity prediction was performed using ProTox-III. ISO exhibited a predicted acute oral LD50 value of 1180 mg/kg, indicating a relatively low acute toxicity risk (Supplementary Table S6). Notably, ISO showed no predicted hepatotoxicity, cardiotoxicity, nephrotoxicity, carcinogenicity, or cytotoxicity, suggesting the absence of major toxicity liabilities. Although several computational models indicated potential signals associated with neurotoxicity, immunotoxicity, and mutagenicity, these predictions require further experimental validation.

## 3. Discussion

Skeletal muscle atrophy is a progressive disorder involving complex molecular mechanisms, and effective therapeutic strategies remain limited. Recent studies have suggested that CCR5 signaling may play an important role in regulating muscle pathological processes [12,13]; however, natural small-molecule CCR5 inhibitors remain poorly characterized. In this study, we combined computational screening and experimental validation approaches to identify a potential natural CCR5-targeting compound and explore its protective effects against skeletal muscle atrophy.

The CCR5 binding pocket intrinsically favors small-molecule ligands characterized by a “cationic center–hydrophobic scaffold–aromatic ring system” architecture [24]. Structurally, the pocket is composed of acidic amino acid residues, hydrophobic cavities, and aromatic interaction sites, which together enable stable binding of ligands possessing positively charged moieties and a certain degree of conformational rigidity [14,25]. Alkaloids generally contain protonatable nitrogen-containing heterocycles, hydrophobic aromatic scaffolds, and relatively rigid spatial conformations, making their structural and physicochemical properties highly compatible with the ligand-recognition features of CCR5 [15,26]. This compatibility not only facilitates the formation of salt bridges, π–π stacking, and hydrophobic interactions, but also enhances ligand stability within the transmembrane binding pocket, thereby improving binding affinity and receptor modulatory activity toward CCR5 [25]. Therefore, alkaloids are regarded as a highly promising class of natural small-molecule scaffolds for CCR5-targeted ligands. Based on these structural considerations, further molecular docking analysis was performed to determine whether the identified candidate could adopt a CCR5-binding mode similar to known antagonists. Previous structural studies have shown that MVC binds deeply within the transmembrane cavity of CCR5 and stabilizes the receptor in an inactive conformation through interactions with residues distributed across helices II, III, V, VI, and VII [15]. Consistent with these observations, the comparable binding pattern of ISO suggests that it may occupy a similar antagonist-binding pocket within CCR5 and potentially function as a natural CCR5 antagonist at the molecular level. Notably, Glu283 has been identified as a critical residue involved in ligand recognition and CCR5 antagonism, and alterations in its protonation state can markedly influence receptor conformation and ligand interaction profiles [27]. Our docking analysis showed that ISO formed stable hydrogen bonds with Glu283 and Tyr251, indicating that these interactions may contribute to stabilization of the inactive conformation of CCR5 and suppression of receptor activation.

Previous studies on anibamine, a natural alkaloid identified as a CCR5 antagonist, have highlighted the importance of positively charged nitrogen-containing moieties and hydrophobic side chains in mediating CCR5 binding [28]. Comparative docking analyses further suggested that natural alkaloids may share conserved interaction characteristics with classical CCR5 antagonists despite possessing structurally distinct scaffolds [28]. Consistent with these observations, ISO contains multiple nitrogen-containing functional groups and a flexible bisbenzylisoquinoline scaffold, which may facilitate stable occupation of the CCR5 binding cavity through coordinated hydrogen-bonding and hydrophobic interactions.

Although molecular docking provides valuable insights into ligand–CCR5 interactions, docking scores alone cannot fully predict biological activity due to limitations of scoring functions. Therefore, experimental validation will be required to further confirm the inhibitory activity of the identified candidates.

To further evaluate whether the predicted ligand–receptor interactions could be maintained under dynamic conditions, molecular dynamics simulations were subsequently performed. Our study demonstrates that ISO forms a stable and well-equilibrated complex with CCR5 throughout the molecular dynamics simulations. Both systems remained structurally stable over the 200 ns production simulations, but ISO consistently showed smaller backbone, complex, and ligand RMSD values than MVC. The low RMSF values observed in the transmembrane helices and ligand-binding pocket indicate reduced structural flexibility in the core functional region of CCR5 upon ISO binding. In contrast, higher fluctuations were mainly localized to the N-/C-terminal regions and intracellular/extracellular loops, which are known to exhibit intrinsic conformational flexibility in GPCR proteins [29]. Notably, the transmembrane core and ligand-binding pocket maintained consistently low mobility throughout the simulation, suggesting that ISO engages in stable and persistent interactions with CCR5 at the binding site. The hydrogen-bond analysis further supported the greater persistence of the ISO–CCR5 interaction. ISO maintained approximately one to three hydrogen bonds with CCR5 during most of the simulation, whereas MVC formed fewer persistent hydrogen bonds. These results suggest that ISO establishes a more stable polar interaction network within the CCR5 binding pocket. In addition, the stable radius of gyration and the absence of significant global conformational drift indicate that ISO binding does not induce large-scale unfolding or destabilization of CCR5. Instead, the protein maintains a compact and structurally conserved architecture during complex formation. The principal component analysis further supports this observation, revealing a stable anisotropic shape without abrupt transitions or directional expansion, consistent with a stable bound state. The presence of a dominant low-energy basin in the free energy landscape further confirms the thermodynamic stability of the ISO–CCR5 complex.

Collectively, these findings indicate that both ISO and MVC can form stable complexes with CCR5 under the simulated membrane environment. However, compared with MVC, ISO exhibited lower structural deviations, reduced ligand mobility, a more persistent hydrogen-bonding network, and a more compact low-energy conformational ensemble. These combined observations support a more conformationally stable and dynamically persistent interaction between ISO and CCR5.

However, computational analyses alone cannot fully reflect biological activity; therefore, cellular experiments were further conducted to validate the functional effects of ISO on CCR5 signaling. Although CCR5 has recently attracted increasing attention in skeletal muscle–related pathological processes, reports of natural compounds acting as direct CCR5 inhibitors remain scarce. To date, anibamine is one of the very few natural alkaloids reported to directly antagonize CCR5, exhibiting an IC_50_ of approximately 1 μM in a competitive binding assay [30]. However, the reported IC_50_ value for anibamine was derived from a gp120/CCR5 competitive binding system, which differs substantially from the present study based on CCL11-induced CCR5 activation and intracellular cAMP responses. Therefore, direct comparison between these IC_50_ values should be made with caution. Moreover, previous studies on natural CCR5-targeting alkaloids have primarily focused on receptor-binding activity or regulation of CCR5 expression in non-muscle cells, whereas their potential roles in skeletal muscle biology remain largely unexplored. For instance, neferine was shown to suppress CCR5 and CCL5 expression under pathological conditions in endothelial cells, but neither direct functional antagonism of CCR5 nor quantitative IC_50_ evaluation was investigated [31].

In our study, ISO restored the reduction in intracellular cAMP levels induced by CCL11 stimulation and exhibited stronger inhibitory activity toward CCR5-related signaling than MVC, while maintaining low cytotoxicity in C2C12 cells. Collectively, these findings suggest that ISO represents a promising natural CCR5-targeting alkaloid and provide a pharmacological basis for its protective effects against CCL11-induced skeletal muscle atrophy.

Since CCR5 activation has been linked to skeletal muscle wasting, we further investigated whether pharmacological inhibition of CCR5 by ISO could alleviate CCL11-induced myotube atrophy. The effective concentration range of ISO compares favorably with several previously reported alkaloids. Berberine, a widely studied isoquinoline alkaloid, typically exerts anti-atrophic effects in C2C12 myotubes at concentrations ranging from 5 to 20 μM, where it attenuates the elevation of MuRF1 and MAFbx and improves myotube morphology [32]. Similarly, the β-carboline alkaloid norharmane enhances myogenic differentiation and delays muscle aging primarily at 2.5 μM and 5 μM [33]. In contrast, ISO produced marked protective effects at concentrations as low as 0.5 μM and achieved near-complete restoration of myotube morphology at 5 μM, suggesting a comparatively high biological activity.

Comparable observations have been reported for capsaicin, which alleviates cisplatin-induced myotube atrophy at concentrations of 25 and 50 μM [18]. Capsaicin treatment improves myotube diameter and suppresses the expression of MuRF1 and MAFbx, but its effective concentration range is substantially higher than that observed for ISO. Likewise, tomatidine has been shown to activate anabolic signaling pathways and attenuate muscle atrophy at low micromolar concentrations (0.1–3 μM) [34]. Notably, the effective concentration range of ISO observed in the present study is comparable to that reported for tomatidine, supporting the high biological activity of ISO in protecting myotubes against atrophic stimuli. It should be noted that several of these compounds, including berberine and capsaicin, have also been reported to exert anti-inflammatory and antioxidant activities through distinct molecular mechanisms [18,32]. Therefore, the lower effective concentration of ISO observed in the present study cannot be solely attributed to these shared pharmacological properties. Instead, it may reflect differences in chemical structure, target interactions, or cellular pharmacodynamics. The underlying basis for its relatively high potency remains to be further investigated.

Based on our previous findings, CCL11 promotes skeletal muscle atrophy by activating CCR5, which triggers α-actin dissociation and ubiquitin-mediated degradation. In this study, ISO effectively mitigated CCL11-induced myotube atrophy through CCR5 inhibition. These results suggest that the anti-atrophic effect of ISO may, at least in part, be attributed to the suppression of CCR5-mediated α-actin dissociation and degradation, thereby preserving myofiber structural integrity.

Moreover, ISO has been reported to exert potent anti-inflammatory effects by reducing the production of pro-inflammatory mediators, including TNF-α, IL-1β, and IL-6 [35,36]. Accordingly, ISO may protect against muscle atrophy by attenuating inflammatory signaling and the inflammation-induced activation of the ubiquitin–proteasome system. In addition, ISO has demonstrated antioxidant activity by scavenging reactive oxygen species, enhancing cellular antioxidant defenses, preserving mitochondrial function, and reducing mitochondrial apoptosis [37,38]. Given the close association of oxidative stress and mitochondrial dysfunction with skeletal muscle atrophy, these mechanisms likely contribute further to the anti-atrophic effects of ISO. Taken together, these findings indicate that ISO may alleviate muscle wasting not only through direct inhibition of CCR5 signaling but also via coordinated regulation of inflammatory responses, oxidative stress, and mitochondrial homeostasis.

Beyond its biological activity, the pharmacological properties of ISO should also be considered for its potential translational application. SwissADME analysis suggested that ISO possesses acceptable physicochemical properties and a generally favorable predicted ADME profile. However, the relatively high lipophilicity and limited predicted aqueous solubility may represent potential challenges affecting its bioavailability and in vivo pharmacological performance [39]. Future studies should aim to enhance its solubility and tissue-targeting efficiency through crystal form optimization and the development of advanced drug delivery systems, such as liposomes or nanoparticles, thereby improving its pharmacological efficacy in skeletal muscle [40].

The pharmacokinetic properties of ISO have been reported in previous studies. After intravenous administration in rats (5 mg/kg), ISO showed measurable systemic exposure (AUC_0_–∞ = 1925.22 ± 671.78 ng/mL·h), a relatively long elimination half-life (t_1_/_2_ = 7.88 ± 0.84 h), and extensive distribution (Vd = 32.47 ± 11.79 L/kg) [39]. These findings, together with its favorable CCR5-binding profile and MVC-like interaction pattern, provided additional support for selecting ISO as a candidate for further pharmacological investigation.

Although ISO exhibited minimal cytotoxicity in our experimental system and demonstrated an acceptable preliminary safety profile, its overall safety and molecular selectivity require further investigation. ProTox-III analysis suggested that ISO had no major predicted toxicity liabilities, including organ toxicity, carcinogenicity, or cytotoxicity. However, several potential toxicity-associated signals, such as neurotoxicity, were identified by computational models. These predictions should be interpreted cautiously, as in silico toxicity assessments provide preliminary indications of potential risks but do not directly represent experimentally confirmed toxic effects.

Beyond safety considerations, the pleiotropic biological activities of ISO should also be taken into account. ISO has been reported to exhibit diverse pharmacological effects, including anti-inflammatory, antidiabetic, antioxidant, and anticancer activities [39]. These findings suggest that ISO may regulate multiple biological pathways rather than acting through a single molecular target. Consistently, SwissTargetPrediction analysis revealed that ISO possesses a broader predicted target spectrum than the reference CCR5 antagonist MVC, with several GPCR family members, including dopamine and serotonin receptors, among the top-ranked predicted targets. Although such polypharmacological properties may contribute to the overall pharmacological profile of ISO, further experimental studies are required to determine their functional relevance and potential off-target effects.

Therefore, the CCR5-related activity identified in the present study can be considered a potential mechanism contributing to the biological effects of ISO. Although these findings provide initial insights into ISO-mediated CCR5 modulation, their physiological relevance remains to be validated in vivo. Further animal studies are needed to determine the in vivo efficacy, pharmacokinetic properties, target selectivity, and long-term safety of ISO.

## 4. Materials and Methods

### 4.1. Establishment of CCR5 Pharmacophore Model

The crystal structure of CCR5 (PDB ID: 4MBS) was obtained from the Protein Data Bank (PDB). The 3D structure SDF file for MVC (PubChem CID: 3002977) was downloaded from the PubChem database (https://pubchem.ncbi.nlm.nih.gov/, accessed on 20 November 2025). The active site of CCR5 was defined based on the binding pocket of the co-crystallized ligand MVC using Discovery Studio 2019 Client. LibDock was then employed to redock MVC into the CCR5 binding site. The top-ranked docking pose with the highest LibDock score was selected to automatically generate the pharmacophore model, which was subsequently used for virtual screening of potential CCR5 inhibitors.

### 4.2. Pharmacophore-Based Virtual Screening

First, the database of 7860 alkaloids was established for virtual screening (Supplementary Table S1). The three-dimensional structures of these compounds were obtained from the PubChem database and Alkaloid Natural Product Library (TargetMol, Boston, MA, USA, L6110), accessed on 20 November 2025. First, compounds were obtained from the commercially available Alkaloid Natural Product Library supplied by TargetMol. Second, additional alkaloids were identified through a literature search of compounds previously reported as naturally occurring or biologically active alkaloids. Literature-derived compounds were included only when they were explicitly described as alkaloids, possessed an unambiguous chemical identity, and had a complete chemical structure available in PubChem. Compounds with ambiguous names, undefined compositions, incomplete structures, or unavailable structural records were excluded. Duplicate entries between the TargetMol library and the literature-derived dataset were identified by comparing compound names, PubChem CID and InChIKeys. Only one structure was retained for each unique compound. The best search method was carried out to obtain the ‘hit’ compounds matching the features in the established pharmacophore. Then, the filtered molecules with fitvalues > 1 and RMSD < 2 were used for further molecular docking.

### 4.3. Molecular Docking

The crystal structure of CCR5 (PDB ID: 4MBS) was retrieved from the Protein Data Bank (PDB). Protein preprocessing was performed using PyMOL 2.5 and AutoDockTools 1.5.7. Crystal water molecules, co-crystallized ligands, and other non-protein components were removed, and only standard amino acid residues were retained. Polar hydrogen atoms were added, non-polar hydrogens were merged, Gasteiger charges were assigned, and the structures were converted into PDBQT format for molecular docking. Ligands were processed using Open Babel and AutoDockTools 1.5.7. Hydrogen atoms were added, non-polar hydrogens were merged, Gasteiger charges were assigned, rotatable bonds were defined, and the structures were converted into PDBQT format before docking. The binding pocket was defined based on the crystallographic ligand (Maraviroc, MVC) in the CCR5–MVC complex structure. The coordinates of the co-crystallized ligand were extracted using PyMOL 2.5, and the center coordinates of the docking grid were calculated according to the spatial extent of MVC. A cubic grid box covering the entire ligand-binding cavity was generated using AutoDockTools 1.5.7. The grid parameters were set as follows: center_x = 150.441, center_y = 108.671, center_z = 22.379; size_x = 50 Å, size_y = 50 Å, size_z = 50 Å, with a spacing of 1.0 Å. Molecular docking was subsequently carried out using AutoDock Vina 1.1.2 to evaluate the binding affinities between candidate compounds and CCR5. All candidate compounds were docked under identical conditions. Considering that this study aimed to comparatively evaluate the potential binding capacity of multiple candidate molecules rather than perform induced-fit virtual screening, the CCR5 receptor was treated as a rigid structure while ligand molecules were allowed conformational flexibility during docking. Docking conformations and receptor–ligand interactions, including hydrogen-bond interactions, were visualized and analyzed using PyMOL 2.5.

The reliability of the docking protocol was validated by redocking the co-crystallized ligand MVC into the CCR5 binding pocket. Briefly, MVC was removed from the CCR5–MVC complex structure and subsequently redocked using the same docking parameters applied for candidate compounds. The RMSD between the redocked MVC conformation and the crystallographic MVC pose was calculated using PyMOL.

### 4.4. Molecular Dynamics Simulations

A total of two simulation systems were constructed, including Isoliensinine (ISO)-CCR5 and MVC-CCR5. For each ligand-bound system, the optimal docking pose was combined with the CCR5 receptor to generate the initial receptor–ligand complex, whereas the apo system was constructed by removing the ligand from the corresponding complex. Missing residues were modeled using Swiss-PdbViewer 4.0, and the resulting structures were inspected and prepared in PyMOL 2.5.

Each system was subsequently submitted to CHARMM-GUI (https://charmm-gui.org/?doc=input/ligandrm, accessed on 16 July 2026) Membrane Builder. The receptor orientation in the membrane was determined using PPM 2.0, and the protein was embedded in a phospholipid bilayer composed of 100% POPC. The initial membrane dimensions in the x–y plane were approximately 9.0 × 9.0 nm, while the box length along the z-axis was automatically adjusted according to the solvent thickness. The systems were solvated with TIP3P water molecules. The systems were all neutralized, and an ionic concentration of 0.15 M NaCl salt solution was used to adjust the concentration of the solvent systems. parameterized using the CHARMM36m force field; ligand parameters were generated using CGenFF 5.0.

All-atom molecular dynamics simulations were performed using GROMACS 2020.6. Following energy minimization (5000 steps), each membrane receptor system was equilibrated using the six-step CHARMM-GUI protocol, consisting of three 125 ps stages and three 500 ps stages, corresponding to a total equilibration time of 1.875 ns per system. The restraints on the protein, ligand, and lipid molecules were gradually released during equilibration. During equilibration, the temperature was maintained at 310 K using the velocity-rescaling (V-rescale) thermostat with a coupling time constant of 1.0 ps. After initial constant-volume (NVT) relaxation, pressure equilibration was performed under the NPT ensemble using a semi-isotropic Berendsen barostat. The reference pressure was set to 1 bar with a coupling constant of 5.0 ps, allowing the membrane x–y plane and z-axis to scale independently. Production simulations were subsequently conducted at 310 K and 1 bar for 200 ns per system, switching to the Parrinello–Rahman barostat for rigorous pressure control. Various analyses, including RMSD, RMSF, Gibbs free energy landscape, and radius of gyration (Rg), were performed on the simulation trajectories.

### 4.5. Cell Culture

The mouse C2C12 myoblast cell line was obtained from the National Collection of Authenticated Cell Cultures (accession number: SCSP-505, Shanghai, China). C2C12 cells were cultured in Dulbecco’s Modified Eagle Medium (DMEM) (Gibco™, Grand Island, NJ, USA, C11995500BT) supplemented with 10% fetal bovine serum (Vivacell, Shanghai, China, C04001-500) and 1% penicillin–streptomycin. When cells reached 80–90% confluence, differentiation was induced by replacing the medium with DMEM containing 2% horse serum (Gibco™, 26050088) and 1% penicillin–streptomycin for 4–5 days. Cells were treated with 1 µM CCL11 alone or in combination with ISO (0.05, 0.5, or 5 µM). Following treatment, cells were collected after 48 h for Western blot analysis, whereas immunofluorescence staining was performed after 72 h of treatment.

### 4.6. Cell Viability Assay

Differentiated C2C12 cells were treated with ISO or MVC at concentrations ranging from 0.1 nM to 100 µM for 48 h. Cell viability was assessed using the Cell Counting Kit-8 (Life-iLab, Shanghai, China, AC11L054) according to the manufacturer’s instructions. Briefly, 10 µL of reagent was added per well, incubated at 37 °C for 1.5 h, and absorbance was measured at 450 nm using a microplate reader.

### 4.7. cAMP Accumulation Assay

Differentiated C2C12 cells were equilibrated in serum-free DMEM for 2 h. Cells were pretreated with 500 µM Methylisobutylxanthine (IBMX) (TargetMol, T1713) for 15 min to inhibit cAMP degradation, followed by treatment with varying concentrations of ISO (MedChemExpress, HY-N0770) or MVC (MedChemExpress, Monmouth Junction, NJ, USA, HY-13004) for 30 min. Subsequently, 30 µM Forskolin (FSK, MedChemExpress, HY-15371) and 1 µM Eotaxin/CCL11 (MedChemExpress, HY-P7160) were added and incubated for 15 min, with FSK stimulating cAMP production and CCL11 activating CCR5 to suppress cAMP accumulation. Cells were washed twice with ice-cold PBS, lysed in 200 µL cold lysis buffer per well, and centrifuged at 4 °C, 1000 rpm for 2 min. cAMP levels were quantified using the CST Cyclic AMP XP^®^ Assay Kit (Danvers, MA, USA, Catalog #4339) following the manufacturer’s instructions.

### 4.8. Immunofluorescence

Cells were fixed in 4% paraformaldehyde for 30 min, permeabilized with 0.2% Triton X-100 for 20 min, and blocked with 5% BSA for 60 min at room temperature. Cells were incubated overnight at 4 °C with Anti-MYH primary antibody (Sigma, Darmstadt, Germany, M4276; 1:400), followed by incubation with Goat Anti-Mouse IgG H&L (Alexa Fluor^®^ 488) secondary antibody (Abcam, Cambridge, UK, ab150113; 1:300) for 60 min at room temperature in the dark. Nuclei were counterstained and mounted with DAPI-containing anti-fade medium (Solarbio, Beijing, China, S2110). Images were acquired using a Leica TCS SP8 confocal microscope (Wetzlar, Germany) under AF488 excitation.

### 4.9. Western Blot

Cells were washed with PBS and lysed in cell lysis buffer. Proteins were separated by SDS–PAGE and detected using enhanced chemiluminescence (Clinx, ChemiScope 6100, Shanghai, China). Primary antibodies were applied as follows: CCR5 (Abmart, Shanghai, China, PA5786S; 1:500), MAFbx (Abcam, ab168372; 1:1000), and MuRF1 (Proteintech, Rosemont, IL, USA, 55456-1-AP; 1:2000). HRP-conjugated secondary antibodies were Goat Anti-Mouse IgG(H+L) (Beyotime, Shanghai, China, A0216; 1:5000) and Goat Anti-Rabbit IgG(H+L) (Beyotime, A0208; 1:5000).

### 4.10. Statistical Analysis

Each group included at least three biological replicates (n ≥ 3). Data are presented as mean ± standard deviation (Mean ± SD). The cAMP concentration induced by 30 μM FSK for 30 min was defined as the maximal cAMP level (Max cAMP), whereas the cAMP concentration obtained after stimulation with 30 μM FSK and 1 μM CCL11 for 15 min was defined as the minimal cAMP level (Min cAMP). The cAMP levels of all other treatment groups were normalized according to the following equation:

$$\mathrm{Relative}\;\mathrm{cAMP}\;(\%)\;=\;\frac{\mathrm{Sample}\;\mathrm{cAMP}-\mathrm{Min}\;\mathrm{cAMP}}{\mathrm{Max}\;\mathrm{cAMP}-\mathrm{Min}\;\mathrm{cAMP}}\text{×}100$$

The inhibition rate was calculated as:

CCR5 Inhibition (%) = 100 − Relative cAMP (%)

Dose–response curves were generated by plotting inhibition (%) against the logarithm of ISO or MVC concentrations (log[ISO/MVC]) using GraphPad Prism version 10.4.2. Half-maximal inhibitory concentrations (IC_50_) were determined by nonlinear regression analysis using a four-parameter logistic (4PL) model.

Differences between groups were analyzed using one-way ANOVA followed by Fisher’s least significant difference (LSD) test, or, in cases of unequal variances, Welch ANOVA followed by Dunnett’s T3 post hoc test. A *p*-value < 0.05 was considered statistically significant.

## 5. Conclusions

In this study, CCR5 was selected as the inhibitory target due to its pronounced upregulation in aging-related skeletal muscle atrophy. Given the absence of clinically available agents capable of suppressing CCR5-mediated muscle wasting, we employed a virtual screening approach to evaluate 7860 natural alkaloids. Candidate compounds were ranked based on their binding affinities to the CCR5 active site as determined by AutoDock Vina. Among them, ISO exhibited a low binding energy and formed hydrogen-bond interactions similar to those of the reference CCR5 inhibitor, MVC. Molecular dynamics simulations further confirmed the stability and robustness of ISO–CCR5 interactions. Furthermore, in vitro experiments showed that ISO effectively inhibited CCR5-mediated signaling and alleviated CCL11-induced C2C12 myotube atrophy with low cytotoxicity. These findings suggest that ISO may protect skeletal muscle against atrophic stimuli through CCR5 modulation and provide a potential natural compound candidate for further investigation in muscle wasting-related disorders. Collectively, these findings provide preliminary evidence supporting CCR5-related activity of ISO and represent an initial exploration of its potential in the development of muscle atrophy-related therapeutic candidates.

## Figures and Tables

**Table 1:** Docking interaction results of the top 15 CCR5-binding ligands and MVC.

Protein	Ligand Name	Pubchem CID	Binding Energy (kcal/mol)	Key Amino Acids Involved in Hydrogen Bonding	Experimental Accessibility
CCR5	MVC	3002977	−10.8	Glu-283, Tyr-251	/
	Hodgkinsine	442105	−11.4	Glu-283, Tyr-251	Limited
	Halitulin	136731130	−10.4	Tyr-37, Thr-284	Limited
	ISO	5274591	−10	Glu-283, Tyr-251	Available
	19′-Oxotabernamine	101199788	−10	Tyr-89	Limited
	Accedinisine	101927008	−10	/	Limited
	Manzamines E	102585991	−10	/	Limited
	10-Hydroxyusambarensine	10433813	−10	Glu-283	Limited
	Capsimine	134249	−10	Thr-105	Limited
	Solaverbascine	15559003	−10	Thr-195, Lys-191	Limited
	Chitraline	157105	−10	Trp-86, Met-287	Available
	Gandharamine	162842959	−10	Try-89	Limited
	Araguspongines K	162864412	−10	/	Limited
	veramiline-3-O-β-d-glucopyranoside	162926537	−10	Glu-283, Tyr-251, Asn-258	Limited
	Tiliamosine	163005446	−10	Ser-180	Limited
	Cinchophyllamine	163193865	−10	Tyr-37, Thr-105	Limited

**Table 2:** Inhibitory effects of ISO against CCR5 and its cytotoxicity in C2C12 cells.

Ligand	IC_50_ ^1^ [95% CI]/μM	CC_50_ ^2^ [95% CI]/μM
ISO	1.314 [0.9266–1.890]	>30
MVC	6.670 [4.728–9.411]	>100

^1^ IC_50_: the half maximal inhibitory concentration, defined as the concentration of ISO or MVC required to inhibit 50% of CCR5 activity. ^2^ CC_50_: the half maximal cytotoxic concentration, defined as the concentration of ISO or MVC that causes 50% reduction in C2C12 cell viability.

## Data Availability

The original contributions presented in this study are included in the article/Supplementary Material. Further inquiries can be directed to the corresponding authors.

## Supplementary Materials

The following supporting information can be downloaded at: https://www.mdpi.com/article/10.3390/molecules31162837/s1, Table S1. List of the 7860 natural alkaloids in the initial virtual screening database. Table S2. List of the 1524 hit compounds matching the pharmacophore features. Table S3. Molecular docking scores and binding free energies of the 789 candidate alkaloids to the CCR5 active site. Table S4. Predicted physicochemical properties, ADME profiles, and drug-likeness characteristics of isoliensinine (ISO). Table S5. Top 15 predicted targets of ISO and MVC by SwissTargetPrediction. Table S6. Predicted toxicity profile of ISO using ProTox 3.0.
